# Single non-pharmacological intervention of depression in the elderly with cognitive dysfunction: a systematic review and network meta-analysis

**DOI:** 10.3389/fpsyt.2025.1608616

**Published:** 2025-11-25

**Authors:** Zhengyi Tan, Baiyun Li, Lanxin Su, Lihua Liu, Yao-Chen Chuang

**Affiliations:** 1 Kiang Wu Nursing College of Macau, Macao, Macao SAR, China; 2 Department of Nursing, Hunan Normal University, Changsha, Hunan, China

**Keywords:** cognitive dysfunction, depression, aging, non-pharmacological intervention, network meta-analysis

## Abstract

**Purpose:**

This study was conducted to appraise the comparative efficacy of single non-pharmacological methods on depression for cognitive dysfunction patients utilizing network meta-analysis (NMA) and resolve ambiguities in existing literature to help practitioners accurately determine the efficacy and formulate the optimal therapeutic models.

**Design:**

Systematic Review and Network meta-analysis.

**Methods:**

PubMed, Embase, Cochrane, and Web of Science were searched. The Cochrane Handbook for Systematic Reviews of Interventions was used to assess the risk of bias in the included studies. Two investigators independently undertook data extraction and quality evaluation.

**Result:**

Overall, 26 articles incorporating 10 single non-pharmacological interventions were identified. Compared to control, GAME (SMD = −1.00, 95% CrI = −1.70 to −0.39) and mindfulness (SMD = −0.58, 95% CrI = −0.99 to −0.17) significantly alleviated depressive symptoms. RTBC (SMD = −0.49, 95% CrI = −0.88 to −0.09) and MUSIC (SMD = −0.47, 95% CrI = −0.84 to −0.08) showed moderate effects, and PE (SMD = −0.37, 95% CrI = −0.67 to −0.09) showed small effects.

**Conclusion:**

In this network meta-analysis, we synthesized 26 trials to quantify the isolated impact of 10 single non-pharmacological interventions on depressive symptoms. Against usual care (basic medical support, sham stimulation, or wait-list), GAME and mindfulness produced the largest and statistically credible reductions. Reminiscence-therapy-based care (RTBC) and music therapy (MUSIC) generated medium benefits, whereas physical exercise (PE) yielded a small yet significant effect. These findings were robust across both direct and indirect evidence, underscoring GAME and mindfulness as the most effective stand-alone non-pharmacological options for mitigating depression.

**Systematic Review Registration:**

https://www.crd.york.ac.uk/prospero/, identifier CRD42024517077.

## 1 Introduction

Dementia represents a serious global health challenge, affecting more than 55 million people worldwide—a figure projected to nearly triple by 2050 (1, 2). Mild cognitive impairment (MCI), a precursor to dementia, has a high prevalence of about 15.4% among older adults in countries such as China and increases the risk of developing Alzheimer’s disease (3, 4). The clinical manifestation is frequently complicated by depressive symptoms. Approximately 32% of patients with dementia also experience depressive symptoms, and another 16% present with comorbid major depressive disorder (5). This bidirectional and pernicious relationship, where depression accelerates cognitive decline and cognitive impairment exacerbates depression, significantly reduces quality of life (6) and elevates mortality rates while also increasing distress, burden, and depression in caregivers (7).

Current first-line pharmacological treatments for depression in dementia patients, such as selective serotonin reuptake inhibitors (SSRIs) and mirtazapine, show limited efficacy and substantial risks (8). A meta-analysis indicates that no single antidepressant (e.g., SSRIs, mirtazapine, venlafaxine) outperforms usual care in treating depression among older adults with cognitive impairment (5). Moreover, these agents carry substantial risks. For example, tricyclics are associated with orthostatic hypotension, anticholinergic effects, and fall risks, while SSRIs increase the risk of hyponatremia, gastrointestinal bleeding, and prolonged QT intervals (particularly with citalopram). These limitations highlight the pressing need to develop safer, more effective non-pharmacological alternatives (9).

Non-pharmacological interventions are recommended as the first-line approach by major clinical guidelines due to their favorable safety profiles and potential to address the multifaceted nature of the condition (9, 10). Such interventions, including RTBC (Reminiscence therapy-based care program) (11), rTMS (repetitive transcranial magnetic stimulation), EA (electro-acupuncture) (12), CE (creative expression) (13), PE (physical exercise), CT (cognitive therapy) (14), MUSIC (music therapy), GAME (game training) (15), AAA (animal-assisted interventions) (16), mindfulness, and other forms, aim to improve patients’ emotional state, enhance mental health, and restore cognitive function by improving brain neuroplasticity.

Despite a compelling theoretical foundation and recognition in clinical guidelines, the evidence for non-pharmacological interventions in alleviating depression among older adults with cognitive dysfunction remains fragmented and inconclusive. Most randomized controlled trials (RCTs) have evaluated non-pharmacological interventions, with few head-to-head comparisons to determine their relative efficacy (15, 17). Previous systematic reviews and meta-analyses have obscured, rather than clarified, the comparative efficacy of single non-pharmacological interventions for depression (5, 18). They have often mixed heterogeneous or multicomponent non-pharmacological interventions, conflating distinct psychological, social, and physical mechanisms, thus obscuring the efficacy of single modalities.

To address this gap, we performed a systematic review and network meta-analysis (NMA) of RCTs evaluating single interventions for depressive symptoms in older adults with cognitive impairment. NMA, an advanced statistical technique, enables indirect comparisons across interventions by integrating direct and indirect evidence to assess relative effects without integrating head-to-head trials (19), overcoming the limitations of prior reviews that lacked ranked efficacy estimates for distinct, single-component interventions. This study elucidates the comparative effectiveness of single non-pharmacological approaches in dementia-related depression, providing evidence-based guidance for precise, individualized clinical strategies.

## 2 Methods

This study followed the PRISMA-2020 guidelines and the extension statement for NMA (PRISMA-NMA) (20) and the Cochrane Handbook for the Systematic Review of Interventions (21). The NMA was preregistered at PROSPERO (CRD42024517077).

### 2.1 Data sources and searches

Publications were retrieved through PubMed, Embase, Cochrane, and WOS from 1 Jan 2010 to 4 Sep 2025, without language restriction, using MeSH words and free words. This timeframe was selected to mitigate potential methodological heterogeneity that could arise from earlier studies, as research approaches and standards in this field have evolved significantly over the past two decades. The search strings in keywords involved study population (elderly, Cognitive Impairment), outcome (Depression), and study types (randomized controlled trials [RCTs]). The search strategy was personalized for each database. Detailed search strategies in PubMed are shown in **Supplementary Table 1**.

### 2.2 Study selection

EndNote 20 was employed to remove duplicate records, followed by primary screening of titles and abstracts and review of full-text documents. Study design and setting, baseline demographics of participants, specific information about the intervention, and reported outcomes were independently extracted by two reviewers. Discrepancies were tackled via discussion. The reference lists of potentially eligible publications were also screened.

The trials were selected based on the PICOS principles: 1) Population: the older adults (average age ≥ 60 years old) diagnosed with cognitive dysfunction (i.e., MCI or dementia from mild to severe). Patients with severe physical or mental comorbidities were excluded. 2) Interventions: Single non-pharmacological interventions, defined as structured, therapeutic modalities (e.g., physical exercise [PE], music therapy, mindfulness) delivered as standalone treatments. 3) Comparisons: The controls received usual care, sham intervention, or did not receive any treatment to ensure a single mode of intervention. 4) Outcomes: Depression was measured using five validated scales: Cornell Scale for Depression in Dementia (CSDD), Beck Depression Inventory (BDI), Geriatric Depression Scale (GDS), Self-rating Depression Scale (SDS), and Neuropsychiatric Inventory–Clinician Rating Scale (NPI-CR). Each scale employed its own response format and scoring system, where higher scores indicated more pronounced depressive symptoms. To enable pooled analysis, we converted all total scores into standardized mean differences (SMD) using the Hedges g formula, with negative values indicating greater symptom reduction. 5) Study design: RCTs, regardless of blinding and publication status. Non-RCTs were excluded. Besides, animal experiments, case reports, individual cases, research advances, conference articles, expert experience, and duplicates were excluded. Studies were not eliminated based on the duration of intervention or follow-up, nor were they restricted by language.

### 2.3 Risk of bias (quality) assessment

The Cochrane Collaboration risk-of-bias 2 (RoB 2) tool was used to assess the risk of bias in the included RCTs (22). The tool comprised five domains through which bias can be introduced (1): bias arising from the randomization process; (2) bias from deviations from intended interventions; (3) bias from missing outcome data; (4) bias in measurement of the outcome; and (5) bias in selection of the reported result. Each domain was evaluated with response options of “yes,” “probably yes,” “probably no,” “no,” or “no information,” and domain-level judgments subsequently informed an overall risk-of-bias rating of “low risk,” “some concerns,” or “high risk of bias”.

Study quality was assessed independently by two authors; and disagreements were resolved through discussion, with a third author consulted when consensus could not be reached.

### 2.4 Data extraction

The author, country, publication year, basic features of participants (stage of dementia, severity of depression), interventions (type, frequency, duration, and total sessions), and outcome measurement tools were extracted from each included RCT. A standardized, pre-piloted form was developed. Before formal use, two independent reviewers tested the form on three randomly selected trials; ambiguous items were discussed, reworded, and consolidated to improve clarity and completeness. The final version was then applied to all included studies.

### 2.5 Statistical analysis

#### 2.5.1 Data synthesis and heterogeneity assessment

All analyses were performed in R (version 4.3.2) using the gemtc package for Bayesian NMA. Due to varying depression assessment scales across studies, SMDs with 95% confidence intervals (CIs) were calculated as the pooled effect size, interpreted as small (0.2), moderate (0.5), or large (0.8) effects (23). Heterogeneity was quantified using the I² statistic, categorized as none (0%), low (25%), moderate (50%), or high (75%). A sensitivity analysis based on pairwise meta-analysis was conducted to further explore the sources and impacts of heterogeneity.

#### 2.5.2 NMA

A Bayesian random-effects NMA was conducted to compare the efficacy of single non-pharmacological interventions, integrating direct and indirect evidence. Interventions were ranked by surface under the cumulative ranking curve (SUCRA) values (24). Model convergence was verified using Gelman-Rubin diagnostics, with simulations employing four chains, 25,000 iterations (first 5,000 discarded), and a deviance information criterion (DIC) for model fit. Local inconsistency was assessed via node-splitting, with DIC differences <5 indicating consistency. To obtain more robust results, we adopted the random-effects model for NMA. The Bayesian model used default vague priors in the gemtc R package: relative treatment effects followed a normal distribution with a mean of 0 and a variance of (15 × 1.04)^2. The standard deviation of between-study heterogeneity followed a uniform distribution of 0 to 1.04, where 1.04 was automatically determined from the data and represented a significant difference on the outcome scale.

#### 2.5.3 Subgroup and sensitivity analyses

Subgroup analyses were performed to explore the sources of heterogeneity, stratified by depression assessment instrument, baseline depression severity, and dementia stage; between-subgroup differences were tested using χ² (p > 0.05 indicating minimal explanatory variance). Scales with limited trials (e.g., SDS [k=1]; NPI-C [k=1]) were excluded from subgrouping. Sensitivity analyses were stratified by primary instrument and excluded high-risk-of-bias studies to evaluate robustness.

#### 2.5.4 Publication bias and small-study effects

Publication bias was evaluated using comparison-adjusted funnel plots, plotting study effect sizes against standard errors for each intervention versus control. Asymmetry was tested with network-specific Egger’s regression (multi-level structure; p > 0.10 indicating no small-study effects). Standard funnel plots supplemented pairwise meta-analyses of interventions versus usual care.

## 3 Result

### 3.1 Identification of relevant studies

**Figure 1:**
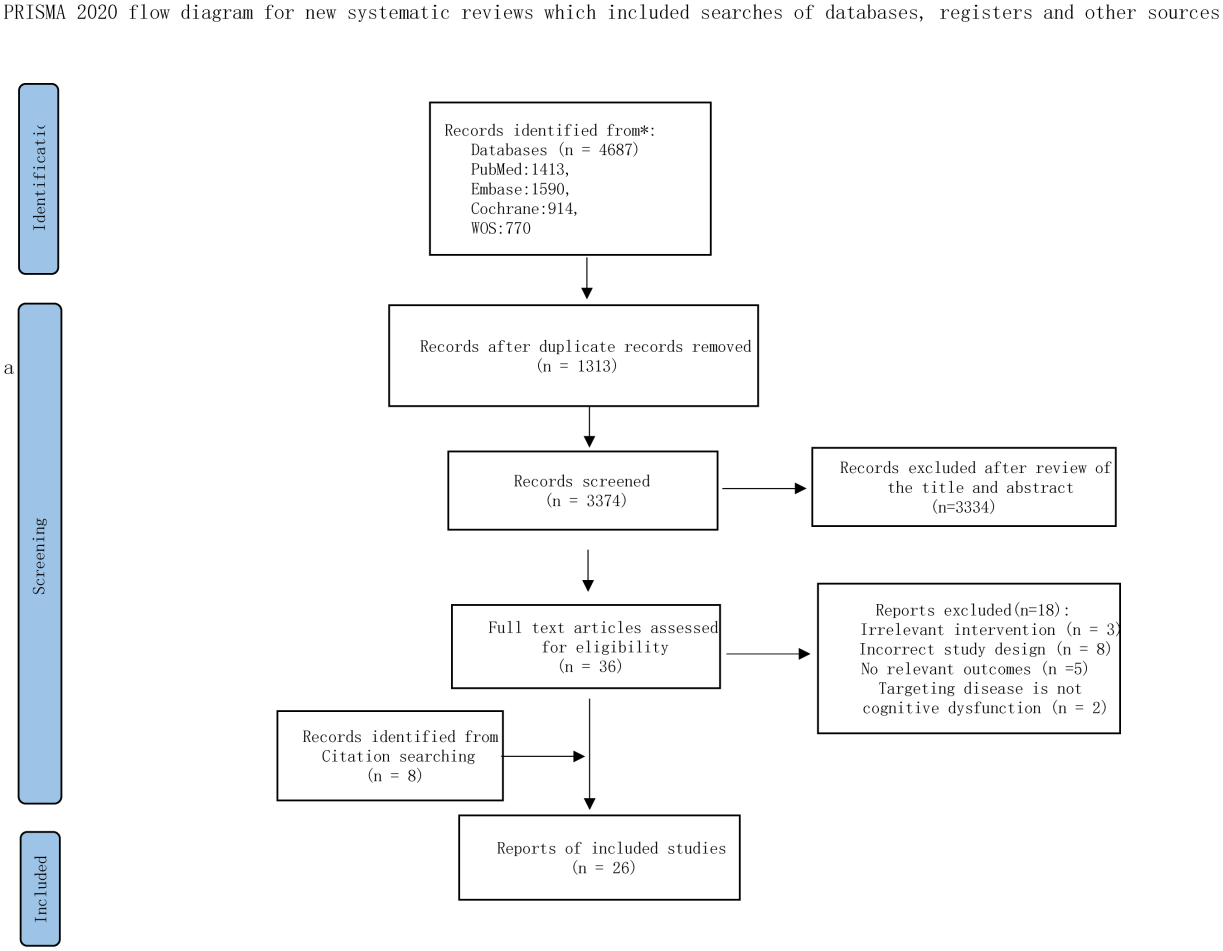
The PRISMA flowchart of selection process. Adapted from Page MJ, McKenzie JE, Bossuyt PM, Boutron I, Hoffmann TC, Mulrow CD, et al. The PRISMA 2020 statement: an updated guideline for reporting systematic reviews. BMJ 2021;372:n71. doi:10.1136/bmj.n71. Licensed under CC BY 4.0.

Following PRISMA guidelines (20), our search yielded 4687 records. After removal of duplicates and screening of titles and abstracts, 36 full-text articles were assessed for eligibility, among which 18 were excluded for not meeting the inclusion criteria, and 8 were excluded due to inaccessible full texts. In addition, 8 studies were identified through the reference lists of relevant reviews. Ultimately, 26 RCTs were included in the NMA, encompassing 1893 participants and 10 distinct single non-pharmacological interventions (**Figure 1**). Of these, 25 RCTs were published in English and one in Spanish.

### 3.2 Characteristics of the included RCTs

The 26 RCTs were conducted between 2010 and 2022, with 1893 participants in 11.

countries (Spain, China, Korea, Singapore, Sweden, Japan, Turkey, Italy, Brazil, Norway, and Greece). The participants were mostly aged 60–80 years old, and the commonly applied diagnostic criteria encompassed the Montreal Cognitive Assessment, Mini-Mental State Examination, and the DSM. Different countries and regions may follow revised guidelines or standards that they have recognized. The severity of cognitive dysfunction ranged from mild to severe.

Interventions included RTBC (25–27), rTMS (12, 28), EA (29), CE (13), PE (30–33), CT (34–40), MUSIC (41–43), GAME (15), AAA (44), and Mindfulness (17, 45, 46). Controls received usual care, health education, sham interventions, placebo, or no treatment. Intervention durations ranged from 1.5 to 10 months (e.g., twice weekly for 6–12 weeks for RTBC, MUSIC, and AAA; 5 times weekly for 2 weeks for rTMS). Depression was assessed using GDS/GDS-15 (k=15), CSDD (k=6), BDI (k=3), SDS (k=1), and NPI-CR (k=1). Detailed characteristics are listed in **Table 1**.

**Table 1:** League table: Relative effects of different interventions.

CTRL
0.49 (0.09, 0.88)	RT
0.16 (-0.48, 0.78)	-0.33 (-1.07, 0.41)	rTMS
0.2 (-0.36, 0.76)	-0.3 (-0.97, 0.4)	0.04 (-0.81, 0.89)	EA
-0.01 (-0.62, 0.58)	-0.51 (-1.22, 0.22)	-0.18 (-1.04, 0.7)	-0.21 (-1.03, 0.6)	CE
0.37 (0.09, 0.67)	-0.13 (-0.6, 0.39)	0.2 (-0.48, 0.92)	0.16 (-0.45, 0.82)	0.38 (-0.27, 1.06)	PE
0.23 (-0.01, 0.5)	-0.26 (-0.71, 0.23)	0.07 (-0.6, 0.76)	0.04 (-0.57, 0.66)	0.25 (-0.39, 0.92)	-0.13 (-0.52, 0.25)	CT
0.47 (0.08, 0.84)	-0.02 (-0.57, 0.52)	0.31 (-0.43, 1.04)	0.27 (-0.42, 0.94)	0.49 (-0.23, 1.18)	0.1 (-0.4, 0.56)	0.23 (-0.25, 0.67)	MUSIC
1.03 (0.39, 1.69)	0.54 (-0.2, 1.32)	0.87 (-0.01, 1.8)	0.83 (-0.01, 1.7)	**1.05 (0.17, 1.95)**	0.66 (-0.05, 1.38)	**0.79 (0.1, 1.5)**	0.56 (-0.17, 1.34)	GAME
0.66 (-0.07, 1.39)	0.17 (-0.66, 1.02)	0.5 (-0.45, 1.48)	0.46 (-0.46, 1.38)	0.67 (-0.3, 1.62)	0.3 (-0.5, 1.07)	0.43 (-0.36, 1.2)	0.2 (-0.64, 1.04)	-0.38 (-1.35, 0.61)	AAA
0.58 (0.17, 0.99)	0.09 (-0.47, 0.67)	0.41 (-0.32, 1.18)	0.37 (-0.31, 1.08)	0.6 (-0.12, 1.32)	0.21 (-0.3, 0.71)	0.34 (-0.14, 0.82)	0.11 (-0.44, 0.68)	-0.45 (-1.23, 0.31)	-0.09 (-0.93, 0.76)	Mindfulness

CTRL, control; RT, Reminiscence therapy; rTMS, repetitive transcranial magnetic stimulation; EA, electro-acupuncture; CE, creative expression; PE, physical exercise; CT, cognitive therapy; MUSIC, music therapy; GAME, game training;, AAA, animal-assisted interventions. Data are Standardized Mean Difference (SMD) with 95% Confidence Intervals (CI). Comparisons are for the column-defining intervention versus the row-defining intervention. Bolded results indicate a statistically significant difference between the two interventions (i.e., the 95% CI does not include 1.0 for ratio measures or 0 for difference measures).

### 3.3 Risk of bias assessment

Risk of bias was evaluated using the RoB 2 tool (22).

**Figure 2:**
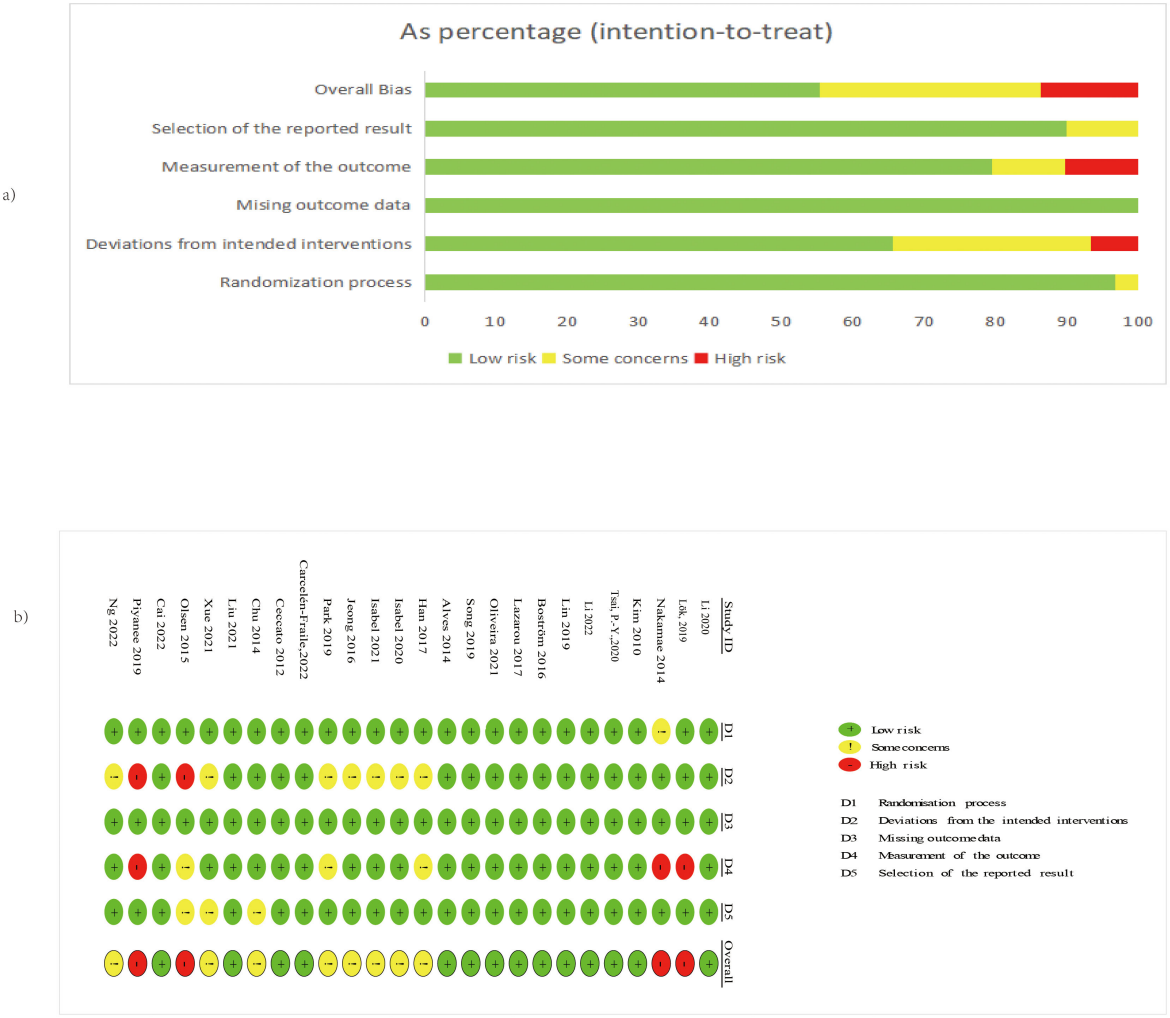
**(A)** The overall risk of bias for all included studies; **(B)** The risk of bias for each study. Adapted from Page MJ, McKenzie JE, Bossuyt PM, Boutron I, Hoffmann TC, Mulrow CD, et al. The PRISMA 2020 statement: an updated guideline for reporting systematic reviews. BMJ 2021;372:n71. doi:10.1136/bmj.n71. Licensed under CC BY 4.0.

Overall, 53.8% of studies (n = 14) had low risk, 30.8% (n = 8) raised some concerns, and 15.4% (n = 4) had high risk (**Figure 2**).

Domain-specific risks were predominantly low: randomization process (96.2% low), deviations from intended interventions (65.4% low), missing outcome data (100% low), measurement of the outcome (76.9% low), and selection of the reported result (88.5% low). High-risk studies were identified in the domains of deviations from interventions and outcome measurement. High-risk studies were Olsen et al. (2016) (domains D2) (27), (domains D4), Lök et al. (26) (domains D4), and (46) (domains D2 and D4); concerns primarily involved blinding and reporting (**Figure 2**) As shown in **Supplementary Figure 1**, the exclusion of high-risk studies did not materially alter the pooled effect estimates.

### 3.4 Results of NMA

The NMA included 26 RCTs comparing 10 interventions against the control. Global heterogeneity was low (I² = 2%) (**Supplementary Figure 2**).

**Figure 3:**
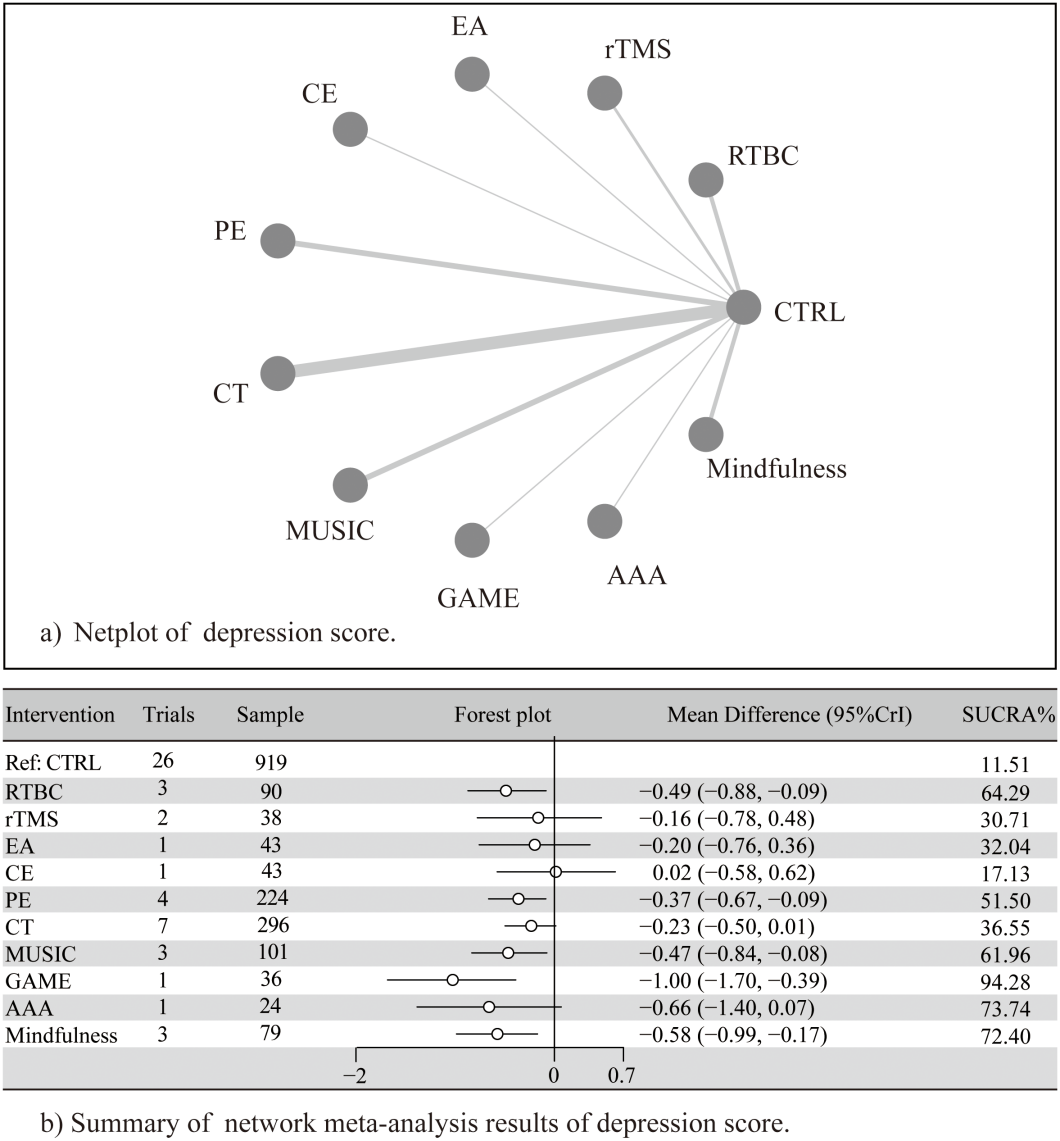
**(A)** Netplot of depression score; **(B)** Summary of network meta-analysis of depression score. Adapted from Page MJ, McKenzie JE, Bossuyt PM, Boutron I, Hoffmann TC, Mulrow CD, et al. The PRISMA 2020 statement: an updated guideline for reporting systematic reviews. BMJ 2021;372:n71. doi:10.1136/bmj.n71. Licensed under CC BY 4.0.

Compared to control, GAME (SMD = −1.00, 95% CrI = −1.70 to −0.39), and mindfulness (SMD = −0.58, 95% CrI = −0.99 to −0.17) significantly alleviated depressive symptoms. RTBC (SMD = −0.49, 95% CrI = −0.88 to −0.09) and MUSIC (SMD = −0.47, 95% CrI = −0.84 to −0.08) showed moderate effects, and PE (SMD = −0.37, 95% CrI = −0.67 to −0.09) showed small effects. AAA (SMD = −0.66, 95% CrI = −1.40 to 0.07), rTMS (SMD = −0.16, 95% CrI = −0.78 to 0.48), EA (SMD = −0.20, 95% CrI = −0.76 to 0.36), CE (SMD = 0.02, 95% CrI = −0.58 to 0.62), and CT (SMD = −0.23, 95% CrI = −0.50 to 0.01) showed non-significant effects (**Figure 3** and **Supplementary Figure 3**-**9**).

The league table also revealed significant differences (**Table 2**). GAME outperformed CE (SMD = 1.05, CrI =0.17 to 1.95), and CT SMD = 0.79, CrI =0.1 to 1.5); SUCRA rankings indicated the following probability order for intervention efficacy: GAME (94.3%), AAA (73.7%), Mindfulness (72.4%), RTBC (64.3%), MUSIC (62.0%), PE (51.5%), CT (36.6%), EA (34.6%), rTMS (32.0%), CE (17.1%), and CTRL (11.5%). Notably, although AAA ranked second according to SUCRA, its effect estimate crossed the null line, suggesting uncertainty in its true efficacy and highlighting the need for cautious interpretation of rankings based on limited direct evidence. The ranking of effects of different non-pharmacological interventions and the description of corresponding treatments are detailed in **Table 3** and **Supplementary Figure 10**.

**Table 2:** The detailed description of corresponding treatments.

trt ID	trt description
1	CTRL
2	RTBC
3	rTMS
4	EA
5	CE
6	PE
7	CT
8	MUSIC
9	GAME
10	AAA
11	Mindfulness

Higher SUCRA indicates better-performing treatments

CTRL, control; RTBC, Reminiscence therapy-based care program; rTMS, repetitive transcranial magnetic stimulation; EA, electro-acupuncture; CE, creative expression; PE, physical exercise; CT, cognitive therapy; MUSIC, music therapy; GAME, game training; AAA, animal-assisted interventions

**Table 3:** Basic characteristics of included studies.

Author/year	Age(year)	Region	Participant All (female)	Diagnose criteria	Dementia stage	Duration	TG	Control group (CG)	Outcome	Baseline depression severity
Li, 2020 (25)	TG:83.2 ± 6.7	China	90(40)	1. ①	MCI(MD)	Twice a week for 12 wks.	RT	Routine treatment	CSDD	no to mild depression
	CG:83.5 ± 5.5			2.CDR						
Nakamae, 2014 (27)	TG:84.8 ± 6.9	Japan	36(NI)	MMSE	MCI(MD)	Weekly for six wks.	RT	Routine care (e.g. basic medical monitoring and daily assistance)	CSDD	no to mild depression
	CG:87.2 ± 4.6									
Lök, 2019 (26)	NA	Turkey	60(34)	1.IWG‐2	Mild to moderate dementia	Weekly 8 wks.	RT	Routine care (e.g. basic medical monitoring and daily assistance)	CSDD	moderate depression
				2.MMSE						
Tsai, 2020 (28)	TG:60.1 ± 14.1	Taiwan	41(8)	RBANS	Mild to moderate dementia	5 times per week for 2 wks.	rTMS	Sham Stimulation	BDI	no to mild depression
	CG:57.5 ± 12.3									
Kim, 2010 (12)	TG:60.9 ± 13.1	Korea	18(8)	MMSE	Mild to moderate dementia	5 times per week for 2 wks.	rTMS	Sham Stimulation	BDI	no to mild depression
	CG:66.8 ± 17.2									
Li, 2022 (47)	TG:65.1 ± 7.5	China	120(58)	MoCA	MCI(MD)	3 times per week for 8 wks.	EA	Sham Acupuncture	SDS	no to mild depression
	CG:64.6 ± 8.4									
Lin, 2019 (13)	TG:85.3 ± 5.9	China	91(57)	1. (ICD)-10	MCI(MD)	Twice a week for 6 wks.	CE	Standard Cognitive	CSDD	no to mild depression
	CG:83.5 ± 8.1			2. MMSE						
Boström, 2016 (30)	NA	Sweden	141(76)	1.DSM-IV-TR	Mild to moderate dementia	4 months	PE	Control Activity	GDS	no to mild depression
				2.MMSE						
Song, 2019 (33)	TG:76.2 ± 5.8	China	120(90)	MoCA-C	MCI(MD)	16 wks.	PE	Health Education	GDS	moderate depression
	CG:75.3 ± 6.8									
Oliveira, 2021 (32)	TG:76.3 ± 6.6	Brazil	54(36)	MMSE	Moderate Dementia	3 months	PE	Psycho-education	NPI-C	no to mild depression
	CG:78.4 ± 8.4									
Lazarou, 2017 (31)	TG:65.9 ± 10.8	Greece	129(NI)	Petersen criteria	MCI(MD)	Twice a week for 10 months	PE	Regular lifestyle	BDI	no to mild depression
	CG:67.9 ± 9.5									
Alves, 2014 (34)	TG:79.6 ± 9.1	Spain	17(13)	GDS	Mild to moderate dementia	1.5 months	CT	Brief Intervention	GDS	no to mild depression
	CG:77.7 ± 12.4									
Gomez-Soria, 2020 (36)	NR	Spain	112(94)	MMSE	MCI(MD)	Weekly for 10 wks.	CT	Routine community medical follow-up	GDS-15	no to mild depression
Gomez-Soria, 2021 (37)	TG:71.5 ± 4.8	Spain	29(23)	MMSE	MCI(MD)	Weekly for 10 wks.	CT	Routine community medical follow-up	GDS-15	no to mild depression
	CG:73,9 ± 5.3									
Han, 2017 (38)	TG:73.7 ± 4.8	Korea	43(20)	1.CERAD-K	MCI(MD)	Twice per week for 4 wks.	CT	Routine community medical follow-up	GDS	moderate depression
	CG:74.5 ± 6.4			2.DSM						
Carcelén-Fraile, 2022 (35)	TG:75.4 ± 3.7	Spain	72(48)	1.MMSE	MCI(MD)	12 wks.	CT	Usual Care	GDS	moderate depression
	CG:74.8 ± 3.9			2.MoCA						
Author/year	age(year)	Region	Participant	Diagnose Criteria	dementia stage	Duration	TG	CG	outcome	Baseline Depression Severity
Jeong, 2016 (39)	TG:69.5 ± 7.8	Korea	224(141)	Petersen criteria	MCI(MD)	5 days per week for 12 wks.	CT	Usual Care	GDS-15	no to mild depression
	CG:71.6 ± 6.5									
Park, 2019 (40)	NA	Korea	49(28)	NIA-AA criteria	MCI(MD)	Daily for 12 wks.	CT	Basic follow-up monitoring	GDS-15	no to mild depression
Chu, 2014 (42)	All:82 ± 6.8	Taiwan	100(53)	DSM	MCI(MD)	Weekly for 6 wks.	MUSIC	Usual Care	CSDD	moderate depression
Ceccato, 2012 (41)	TG:85.5 ± 5.9	Italy	50(30)	DSM	Mild to moderate dementia	Twice a week for 12 wks.	MUSIC	Standard care (eg. basic medical monitoring and daily care)	GDS	moderate to high
	CG:87.2 ± 7.1									
Liu, 2021 (43)	TG:86.6 ± 4.5	Taiwan	50(0)	①	Mild to moderate dementia	Weekly for 12 wks.	MUSIC	non-music-related interventions (e.g. rest and reading)	GDS	no to mild depression
	CG:86.9 ± 5.7									
Xue, 2021 (15)	TG:75.4 ± 4.6	China	72(48)	MoCA	MCI(MD)	Three times per week for 8 wks.	GAME	Routine care (e.g. health education and basic daily care)	GDS–15	no to mild depression
	CG:73.4 ± 4.9									
Author/year	age(year)	Region	Participant	Diagnose Criteria	dementia stage	Duration	TG	CG	outcome	Baseline Depression Severity
Olsen, 2015 (43)	TG:82.9 ± 8.5	Norway	51(32)	MMSE	MCI(MD)	Twice a week for 12 wks.	AAA	No animal-assisted intervention	CSDD	no to mild depression
	CG:84.1 ± 6.7									
Piyanee, 2019 (46)	TG:71.3 ± 5.6	Singapore	56(41)	Petersen criteria	MCI(MD)	9 months	Mindfulness	Health Education	GDS	no to mild depression
	CG:71.4 ± 6.0									
Cai, 2022 (17)	TG:80 ± 8.0	China	75(56)	1.MoCA	MCI(MD)	Weekly for 8 wks.	Mindfulness	Health Education	GDS-15	no to mild depression
	CG:80 ± 10.8			2.MMSE						
Ng, 2022 (45)	TG:71.9 ± 5.9	Singapore	55(41)	Petersen criteria	MCI(MD)	9 months	Mindfulness	Health Education	GDS–15	no to mild depression
	CG:70.7 ± 6.2									

①: National Institute of Neurological Communicative Disorders and Stroke–Alzheimer’s Disease and Related Disorders Association criteria

MoCA, Montreal Cognitive Assessment; MMSE, Mini-Mental State Examination; DSM, Diagnostic and Statistical Manual of Mental Disorders;

CDR, Clinical Dementia Rating; RBANS, Assessment of Neuropsychological Status; CERAD-K, Consortium to Establish a Registry for Alzheimer’s Disease; RT, Reminiscence therapy; rTMS, repetitive transcranial magnetic stimulation; EA, electro-acupuncture; CE, creative expression; PE, physical exercise; CT, cognitive therapy; MUSIC, music therapy; GAME, game training;, AAA, animal-assisted interventions CSDD, Cornell Scale for Depression in Dementia; SDS, Self-rating Depression Scale; NPI-C, Neuropsychiatric Inventory–Clinician Rating Scale; GDS, Geriatric Depression Scale; BDI, Beck Depression Inventory; NIA-AA, National Institute on Aging – Alzheimer’s Association criteria; (ICD)-10, World Health Organization (WHO) and International Classification of Disease (ICD)-10. TG, Treatment Group; CG, Control Group.

### 3.5 Publication bias, subgroup analyses, and sensitivity analyses

#### 3.5.1 Publication bias and small-study effects

Funnel plots showed symmetry (**Supplementary Figure 11**), supported by Egger’s test (p=0.43), indicating no evidence of publication bias or small-study effects. Outlying points may reflect study heterogeneity rather than bias.

#### 3.5.2 Subgroup analyses by depression scale

Stratification by primary assessment scales (e.g., GDS, CSDD, BDI) yielded intervention-favoring effects (all 95% CIs excluded zero), with negligible between-subgroup heterogeneity (χ²=0.42, df=2, p=0.81), indicating that different scales minimally affected the results (**Supplementary Figure 1**).

#### 3.5.3 Sensitivity analyses

In the NMA, pairwise comparisons revealed high heterogeneity for MUSIC (I² = 62%) and PE (I² = 68%). To evaluate the robustness of this finding, we conducted leave-one-out sensitivity analyses, sequentially omitting each study from the pairwise SMD estimate. Results indicated that exclusion of any single study did not substantially alter the pooled SMD, suggesting that the observed heterogeneity was acceptable and unlikely driven by a single study. These findings support the stability of the network estimates (**Supplementary Figures S12**-**14**).

## 4 Discussion

This NMA confirms the efficacy of non-pharmacological interventions in alleviating depressive symptoms in older adults with dementia. Four interventions (RTBC, PE, MUSIC, and mindfulness) outperformed the control, with GAME demonstrating superior efficacy across multiple outcome measures, positioning it as a potential first-line therapy. Compared with previous meta-analyses that identified effective combinations of non-pharmacological and pharmacological interventions, such as cognitive stimulation combined with rehabilitation (48) and cognitive stimulation combined with cholinesterase inhibitors (5), for alleviating depressive symptoms in individuals with dementia (predominantly without major depressive disorder), our findings highlight GAME and mindfulness as the most promising single-modality interventions. These differences may be attributed to variations in study populations (our analysis included participants with MCI and mild-to-severe dementia, whereas earlier studies focused exclusively on dementia), intervention scope (single versus combined modalities), and outcome assessment (we incorporated multiple validated scales without prioritizing a specific tool, while Watt et al. emphasized the Cornell Scale for depression in dementia). Such methodological distinctions, while enabling broader evidence synthesis in our study, also contributed to increased variability in comparative results.

### 4.1 Mechanisms of non-pharmacological interventions

For depression in older adults with cognitive impairment, such as MCI and dementia, non-pharmacological interventions exert their effects through multifaceted mechanisms that target cognitive, emotional, and social domains. These approaches often synergistically address neurodegeneration, inflammation, and psychosocial stressors inherent to cognitive impairment, offering safer alternatives to pharmacotherapy, which has shown limited efficacy and notable adverse effects (5, 9).

GAME represents a promising non-pharmacological strategy for mitigating the multifaceted challenges of dementia, particularly by fostering social engagement and emotional resilience. It promotes social interaction, emotional expression, and interpersonal communication among individuals with cognitive impairments, providing opportunities for releasing emotional stress and achieving a sense of accomplishment. In turn, it enhances self-efficacy and subjective well-being, critical psychological buffers that impede dementia-related cognitive decline and alleviate depressive symptoms. By creating structured and enjoyable collaborative environments, games enable older adults to re-establish interpersonal connections, thereby reducing isolation, fostering a renewed sense of agency, and indirectly mitigating the emotional toll caused by cognitive decline (15, 49).

MUSIC, mindfulness, and RTBC cultivate supportive environments that acknowledge personal experiences and emotional narratives, which foster emotional resilience and regulation and produce antidepressant benefits. For instance, MUSIC promotes well-being and reduces depressive symptoms by evoking pleasant memories, fostering peer support, and enhancing self-confidence and a sense of belonging. Empirical evidence confirms its value as a nursing intervention to enhance cognition, quality of life, and mood in Alzheimer’s patients (26, 50–52). Similarly, mindfulness alleviates psychological distress BY promoting present-moment awareness, while RTBC reconstructs positive self-narratives and strengthens interpersonal bonds via reminiscence.

PE, in contrast, exerts its antidepressant effects through robust neurobiological adaptations. It enhances hippocampal neuroplasticity, strengthens antioxidant defenses, and maintains cognitive-emotional homeostasis while suppressing neurodegeneration and inflammation, hallmarks of comorbid dementia and depression. At the molecular level, PE modulates the hypothalamic-pituitary-adrenal (HPA) axis, elevates neurotrophic factors, such as brain-derived neurotrophic factor (BDNF), and attenuates neuroinflammation, collectively improving mood (48, 53, 54).

### 4.2 The superiority of GAME therapy

The superior performance of GAME in this NMA, as evidenced by its large effect size (SMD = −1.00) compared to other interventions, may be attributed to its unique integration of cognitive, physical, and social elements in an engaging gamification design. Unlike single-targeted approaches, such as PE (primarily targets neurobiological pathways) or psychosocial therapies (focuses on emotional validation), GAME incorporates three elements: mental stimulation (e.g., memory and problem-solving tasks), motor activities (e.g., interactive movements in video or board games), and social collaboration (e.g., group games that promote interpersonal bonds). This multifaceted nature may enhance adherence through intrinsic motivation and enjoyment, leading to broader impacts on behavioral and psychological symptoms of dementia, including depression (49). A previous study has demonstrated that incorporating memory games (e.g., remembering the sequence and color of balls), coordination games, and solitaire or board games (e.g., poker, puzzles, Chinese letter games, and number guess games) into game training interventions can significantly reduce depressive symptoms, improve cognition, and enhance subjective well-being. This therapy outperforms usual care due to synergistic effects on brain plasticity, social relationships, and participant engagement ^15^. The entertainment value and adaptability of the GAME therapy make it a versatile tool, which may explain why it can promote continued engagement and holistic symptom relief in dementia populations, thus outperforming other therapies.

### 4.3 Strengths

This NMA advances evidence synthesis on non-pharmacological interventions for depressive symptoms in older adults with cognitive impairment, including MCI and dementia. Integrating 26 RCTs across multiple countries and over 2,000 participants, it comprehensively evaluates 10 single interventions, resolving fragmentation in prior reviews that conflate heterogeneous approaches. Methodological strengths include adherence to PRISMA guidelines, low heterogeneity, symmetrical funnel plots (no publication bias), and high consensus in RoB 2 assessments, with most studies showing low risk in key domains.

The results outline clear efficacy ratings, prioritizing game therapy, music therapy, and mindfulness, which provide person-centered guidance for clinicians, caregivers, and policymakers to tailor strategies based on dementia stage and depression severity. They may be safer alternatives to pharmacotherapy, which have limited efficacy and risks (e.g., SSRIs, tricyclics), providing guideline support for non-pharmacological options.

Future research should emphasize real-world feasibility, including caregiver training, long-term evaluations, and cost-benefit analysis. Personalized interventions for dementia patients with comorbid depression are essential, where funding policies and integration into standard care are critical. Overall, this NMA highlights the potential of single non-pharmacological modalities in enhancing outcomes, easing caregiver burden, and tackling the global dementia crisis through accessible, community-based care.

### 4.4 Limitations

This meta-analysis has several limitations. First, the included studies exhibited heterogeneity in participant cognitive dysfunction levels, ranging from MCI due to Alzheimer’s disease to mild-to-moderate and moderate dementia. This variability may affect the efficacy of non-pharmacological interventions, as cognitive and functional capacities differ across stages. For instance, interventions, like mindfulness and cognitive training, may be more effective for MCI or mild dementia, where cognitive reserve is relatively preserved, but they may require adaptations for moderate dementia. Due to the limited number of studies in each intervention and dementia stage, we could not stratify results by cognitive impairment level, restricting conclusions about optimal interventions for specific stages. Second, variability in intervention protocols (e.g., duration, frequency) and participant characteristics, including dementia stages, limits the generalizability of findings. Additionally, the network geometry was characterized by sparse data for some interventions and heavy reliance on indirect comparisons, which increases uncertainty in efficacy rankings. Furthermore, although the SUCRA values helped to summarize the relative ranking of the interventions, these rankings should be interpreted cautiously, especially for interventions (such as GAME and AAA) that were used by only one small trial. A high SUCRA value does not necessarily indicate confirmed efficacy, particularly when the associated credible intervals are wide or overlap with the null. The restricted scope of interventions focused on structured therapeutic modalities and did not encompass broader lifestyle domains, such as integrated management of physical activity, sedentary behavior, and sleep—areas that are critically linked to mental health in older adults (55–57). Future research should standardize intervention protocols and investigate their efficacy across distinct cognitive impairment stages to enhance clinical applicability. While no evidence of publication bias was detected through funnel plots and statistical tests, the small number of included RCTs constrains the robustness of these assessments. Future research should standardize intervention protocols and investigate their efficacy across distinct cognitive impairment stages to enhance clinical applicability.

## 5 Conclusions

Game therapy, music therapy, and mindfulness are the most effective single non-pharmacological interventions for reducing depressive symptoms relative to controls. Reminiscence therapy-based care provides moderate benefits, and physical exercise offers modest improvements. Methodological limitations call for caution in interpreting the findings, and high-quality RCTs are needed for validation. Future research should validate these findings through larger, direct-comparison trials and expand the comparative framework to include a broader range of lifestyle-oriented interventions.

## Data Availability

The original contributions presented in the study are included in the article/**Supplementary Material**. Further inquiries can be directed to the corresponding authors.

## Supplementary material

Supplementary Table 1 PubMed Search strategy

Supplementary Figure 1 Subgroup analyses by depression scale. **(A)** Subgroup CSDD; **(B)** Subgroup GDS; **(C)** Subgroup BDI

Supplementary Figure 2 Results of network meta-analysis

Supplementary Figure 3 Comparative Analysis between trt 2 versus CTRL

Supplementary Figure 4 Comparative Analysis between trt 3, 4 versus CTRL

Supplementary Figure 5 Comparative Analysis between trt 5 versus CTRL

Supplementary Figure 6 Comparative Analysis between trt 6 versus CTRL

Supplementary Figure 7 Comparative Analysis between trt 7 versus CTRL

Supplementary Figure 8 Comparative Analysis between trt 8 and 9 versus CTRL

Supplementary Figure 9 Comparative Analysis between trt 11 and 12 versus CTRL

Supplementary Figure 10 The figure of ranking probability of reduction of depression scores

Supplementary Figure 11 Funnel plots

Supplementary Figure 12 MUSIC Sensitivity Analyses. **(A)** Intervention position regression; **(B)** Instrument regression; **(C)** Duration meta-regression; **(D)** Dementia stage regression

Supplementary Figure 13 PE sensitivity analyses. **(A)** Duration meta-regression; **(B)** Dementia stage regression; **(C)** Depression severity regression

Supplementary Figure 14 Before and after removing high-risk articles
