# Supplementary material for: Single non-pharmacological intervention of depression in the elderly with cognitive dysfunction: a systematic review and network meta-analysis
Source: Front Psychiatry. 2025 Nov 25;16:1608616. doi: 10.3389/fpsyt.2025.1608616 (PMC12687036; doi:10.3389/fpsyt.2025.1608616)
Supplement: Supplementary Table 1 — PubMed Search strategy [file Supplementaryfile1.docx]

**Supplementary tables**

**Supplementary Table S1: Pubmed Search strategy**

| #1 "Cognition Disorder*"[Title/Abstract] OR "Cognitive Decline*"[Title/Abstract] OR "Cognitive Defect*"[Title/Abstract] OR "Cognitive Deficit"[Title/Abstract] OR "Cognitive Disability"[Title/Abstract] OR "Cognitive Disorder*"[Title/Abstract] OR "Cognitive Dysfunction*"[Title/Abstract] OR "Cognitive Impairment*"[Title/Abstract] OR "Decline*, Cognitive"[Title/Abstract] OR "Delirium, Dementia, amnestic, Cognitive disorders"[Title/Abstract] OR "Deterioration*, Mental"[Title/Abstract] OR "DisorDer*, Cognitive"[Title/Abstract] OR "Dysfunction*, Cognitive"[Title/Abstract] OR "Impairment*, Cognitive"[Title/Abstract] OR "Impairment*, Mild Cognitive"[Title/Abstract] OR "Mental Deterioration*"[Title/Abstract] OR "Mild Cognitive Impairment*"[Title/Abstract] OR "overinclusion"[Title/Abstract] OR "response interference"[Title/Abstract] |
| --- |
| #2 Cognitive Impairment[MeSH Terms] |
| #3 "central depression"[Title/Abstract] OR "clinical depression"[Title/Abstract] OR "depression"[Title/Abstract] OR "Depression, Emotional"[Title/Abstract] OR "depressive disease"[Title/Abstract] OR "depressive disorder"[Title/Abstract] OR "depressive episode"[Title/Abstract] OR "depressive illness"[Title/Abstract] OR "depressive personality disorder"[Title/Abstract] OR "depressive state"[Title/Abstract] OR "Depressive Symptom*"[Title/Abstract] OR "depressive syndrome"[Title/Abstract] OR "Emotional Depression"[Title/Abstract] OR "mental depression"[Title/Abstract] OR "parental depression"[Title/Abstract] OR "Symptom, Depressive"[Title/Abstract] |
| #4 Depression[MeSH Terms] |
| #5 "Aged"[Title/Abstract] OR "elderly"[Title/Abstract] OR "aged patient"[Title/Abstract] OR "aged people"[Title/Abstract] OR "aged person"[Title/Abstract] OR "aged subject"[Title/Abstract] OR "elderly"[Title/Abstract] OR "elderly patient"[Title/Abstract] OR "elderly people"[Title/Abstract] OR "elderly person"[Title/Abstract] OR "elderly subject"[Title/Abstract] OR "senior citizen"[Title/Abstract] OR "senium"[Title/Abstract] |
| #6 Random*[Title/Abstract] OR control*[Title/Abstract] |
| #7 (#1 OR #2) AND (#3 OR #4) AND #5 |
| #8 #6 AND #7 |

**EMBASE Search strategy**

#1 'cognition disorder*':ab,ti,kw OR 'cognitive decline*':ab,ti,kw OR 'cognitive defect*':ab,ti,kw OR 'cognitive deficit':ab,ti,kw OR 'cognitive disability':ab,ti,kw OR 'cognitive disorder*':ab,ti,kw OR 'cognitive dysfunction*':ab,ti,kw OR 'cognitive impairment*':ab,ti,kw OR 'decline*, cognitive':ab,ti,kw OR 'delirium, dementia, amnestic, cognitive disorders':ab,ti,kw OR 'deterioration*, mental':ab,ti,kw OR 'disorder*, cognitive':ab,ti,kw OR 'dysfunction*, cognitive':ab,ti,kw OR 'impairment*, cognitive':ab,ti,kw OR 'impairment*, mild cognitive':ab,ti,kw OR 'mental deterioration*':ab,ti,kw OR 'mild cognitive impairment*':ab,ti,kw OR 'overinclusion':ab,ti,kw OR 'response interference':ab,ti,kw

#2 'cognitive defect'/exp

#3 'aged patient':ti,ab,kw OR 'aged people':ti,ab,kw OR 'aged person':ti,ab,kw OR 'aged subject':ti,ab,kw OR 'elderly':ti,ab,kw OR 'elderly patient':ti,ab,kw OR 'elderly people':ti,ab,kw OR 'elderly person':ti,ab,kw OR 'elderly subject':ti,ab,kw OR 'senior citizen':ti,ab,kw OR 'senium':ti,ab,kw

#4 'aged'/exp

#5 'central depression':ti,ab,kw OR 'clinical depression':ti,ab,kw OR 'depression':ti,ab,kw OR 'depression, emotional':ti,ab,kw OR 'depressive disease':ti,ab,kw OR 'depressive disorder':ti,ab,kw OR 'depressive episode':ti,ab,kw OR 'depressive illness':ti,ab,kw OR 'depressive personality disorder':ti,ab,kw OR 'depressive state':ti,ab,kw OR 'depressive symptom*':ti,ab,kw OR 'depressive syndrome':ti,ab,kw OR 'emotional depression':ti,ab,kw OR 'mental depression':ti,ab,kw OR 'parental depression':ti,ab,kw OR 'symptom, depressive':ti,ab,kw

#6 'depression'/exp

#7 'random*':ti,ab,kw OR 'control*':ti,ab,kw

#8 (#1 OR #2) AND #3 AND (#5 OR #6) AND #7

#9 #8 AND 'Article'/ti

**Web of Science Search strategy**

(TI=(("Cognition Disorder*") OR ("Cognitive Decline*") OR ("Cognitive Defect*") OR ("Cognitive Deficit") OR ("Cognitive Disability") OR ("Cognitive Disorder*") OR ("Cognitive Dysfunction*") OR ("Cognitive Impairment*") OR ("Decline*, Cognitive") OR ("Delirium, Dementia, amnestic, Cognitive disorders") OR ("Deterioration*, Mental") OR ("DisorDer*, Cognitive") OR ("Dysfunction*, Cognitive") OR ("Impairment*, Cognitive") OR ("Impairment*, Mild Cognitive") OR ("Mental Deterioration*") OR ("Mild Cognitive Impairment*") OR ("overinclusion") OR ("response interference")) OR AB=(("Cognition Disorder*") OR ("Cognitive Decline*") OR ("Cognitive Defect*") OR ("Cognitive Deficit") OR ("Cognitive Disability") OR ("Cognitive Disorder*") OR ("Cognitive Dysfunction*") OR ("Cognitive Impairment*") OR ("Decline*, Cognitive") OR ("Delirium, Dementia, amnestic, Cognitive disorders") OR ("Deterioration*, Mental") OR ("DisorDer*, Cognitive") OR ("Dysfunction*, Cognitive") OR ("Impairment*, Cognitive") OR ("Impairment*, Mild Cognitive") OR ("Mental Deterioration*") OR ("Mild Cognitive Impairment*") OR ("overinclusion") OR ("response interference")) OR AK=(("Cognition Disorder*") OR ("Cognitive Decline*") OR ("Cognitive Defect*") OR ("Cognitive Deficit") OR ("Cognitive Disability") OR ("Cognitive Disorder*") OR ("Cognitive Dysfunction*") OR ("Cognitive Impairment*") OR ("Decline*, Cognitive") OR ("Delirium, Dementia, amnestic, Cognitive disorders") OR ("Deterioration*, Mental") OR ("DisorDer*, Cognitive") OR ("Dysfunction*, Cognitive") OR ("Impairment*, Cognitive") OR ("Impairment*, Mild Cognitive") OR ("Mental Deterioration*") OR ("Mild Cognitive Impairment*") OR ("overinclusion") OR ("response interference"))) AND (TI=(("central depression") OR ("clinical depression") OR ("depression") OR ("Depression, Emotional") OR ("depressive disease") OR ("depressive disorder") OR ("depressive episode") OR ("depressive illness") OR ("depressive personality disorder") OR ("depressive state") OR ("Depressive Symptom*") OR ("depressive syndrome") OR ("Emotional Depression") OR ("mental depression") OR ("parental depression") OR ("Symptom, Depressive")) OR AB=(("central depression") OR ("clinical depression") OR ("depression") OR ("Depression, Emotional") OR ("depressive disease") OR ("depressive disorder") OR ("depressive episode") OR ("depressive illness") OR ("depressive personality disorder") OR ("depressive state") OR ("Depressive Symptom*") OR ("depressive syndrome") OR ("Emotional Depression") OR ("mental depression") OR ("parental depression") OR ("Symptom, Depressive")) OR AK=(("central depression") OR ("clinical depression") OR ("depression") OR ("Depression, Emotional") OR ("depressive disease") OR ("depressive disorder") OR ("depressive episode") OR ("depressive illness") OR ("depressive personality disorder") OR ("depressive state") OR ("Depressive Symptom*") OR ("depressive syndrome") OR ("Emotional Depression") OR ("mental depression") OR ("parental depression") OR ("Symptom, Depressive"))) AND (TI=(("aged patient") OR ("aged people") OR ("aged person") OR ("aged subject") OR ("elderly") OR ("elderly patient") OR ("elderly people") OR ("elderly person") OR ("elderly subject") OR ("senior citizen") OR ("senium")) OR AB=(("aged patient") OR ("aged people") OR ("aged person") OR ("aged subject") OR ("elderly") OR ("elderly patient") OR ("elderly people") OR ("elderly person") OR ("elderly subject") OR ("senior citizen") OR ("senium")) OR AK=(("aged patient") OR ("aged people") OR ("aged person") OR ("aged subject") OR ("elderly") OR ("elderly patient") OR ("elderly people") OR ("elderly person") OR ("elderly subject") OR ("senior citizen") OR ("senium"))) AND (TI=(random* OR control*) OR AB=(random* OR control*) OR AK=(random* OR control*))

**Cochrane Library Search strategy**

#1 'cognition disorder*':ab,ti,kw OR 'cognitive decline*':ab,ti,kw OR 'cognitive defect*':ab,ti,kw OR 'cognitive deficit':ab,ti,kw OR 'cognitive disability':ab,ti,kw OR 'cognitive disorder*':ab,ti,kw OR 'cognitive dysfunction*':ab,ti,kw OR 'cognitive impairment*':ab,ti,kw OR 'decline*, cognitive':ab,ti,kw OR 'delirium, dementia, amnestic, cognitive disorders':ab,ti,kw OR 'deterioration*, mental':ab,ti,kw OR 'disorder*, cognitive':ab,ti,kw OR 'dysfunction*, cognitive':ab,ti,kw OR 'impairment*, cognitive':ab,ti,kw OR 'impairment*, mild cognitive':ab,ti,kw OR 'mental deterioration*':ab,ti,kw OR 'mild cognitive impairment*':ab,ti,kw OR 'overinclusion':ab,ti,kw

#2 MeSH descriptor: [Cognitive Dysfunction] explode all trees

#3 ('elderly' OR 'elderly patient' OR 'elderly people' OR 'elderly person' OR 'elderly subject' OR 'senior citizen' OR 'senium'):ti,ab

#4 'central depression':ti,ab,kw OR 'clinical depression':ti,ab,kw OR 'depression':ti,ab,kw OR 'depression, emotional':ti,ab,kw OR 'depressive disease':ti,ab,kw OR 'depressive disorder':ti,ab,kw OR 'depressive episode':ti,ab,kw OR 'depressive illness':ti,ab,kw OR 'depressive personality disorder':ti,ab,kw OR 'depressive state':ti,ab,kw OR 'depressive symptom*':ti,ab,kw OR 'depressive syndrome':ti,ab,kw OR 'emotional depression':ti,ab,kw OR 'mental depression':ti,ab,kw OR 'parental depression':ti,ab,kw OR 'symptom, depressive':ti,ab,kw

#5 MeSH descriptor: [Depression] explode all trees

#6 'random*':ti,ab,kw OR 'control*':ti,ab,kw

#7 (#1 OR #2) AND #3 AND (#5 OR #4) AND #6
