# Supplementary material for: Single non-pharmacological intervention of depression in the elderly with cognitive dysfunction: a systematic review and network meta-analysis
Source: Front Psychiatry. 2025 Nov 25;16:1608616. doi: 10.3389/fpsyt.2025.1608616 (PMC12687036; doi:10.3389/fpsyt.2025.1608616)
Supplement: Supplementary Figure 1 — Subgroup analyses by depression scale. (A) Subgroup CSDD; (B) Subgroup GDS; (C) Subgroup BDI [file Supplementaryfile2.docx]

#### **Supplementary Figures**

| 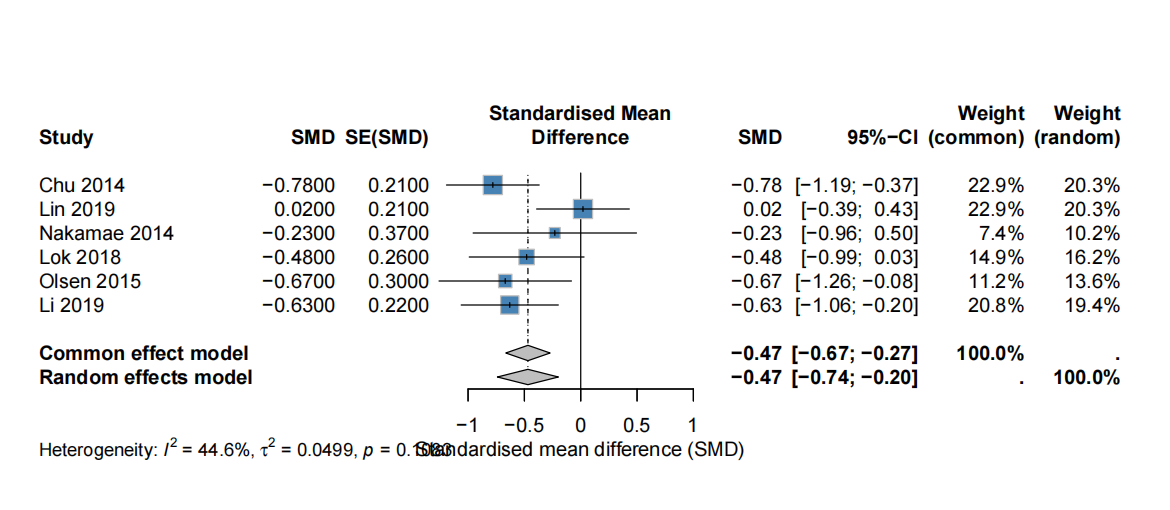  **A** | 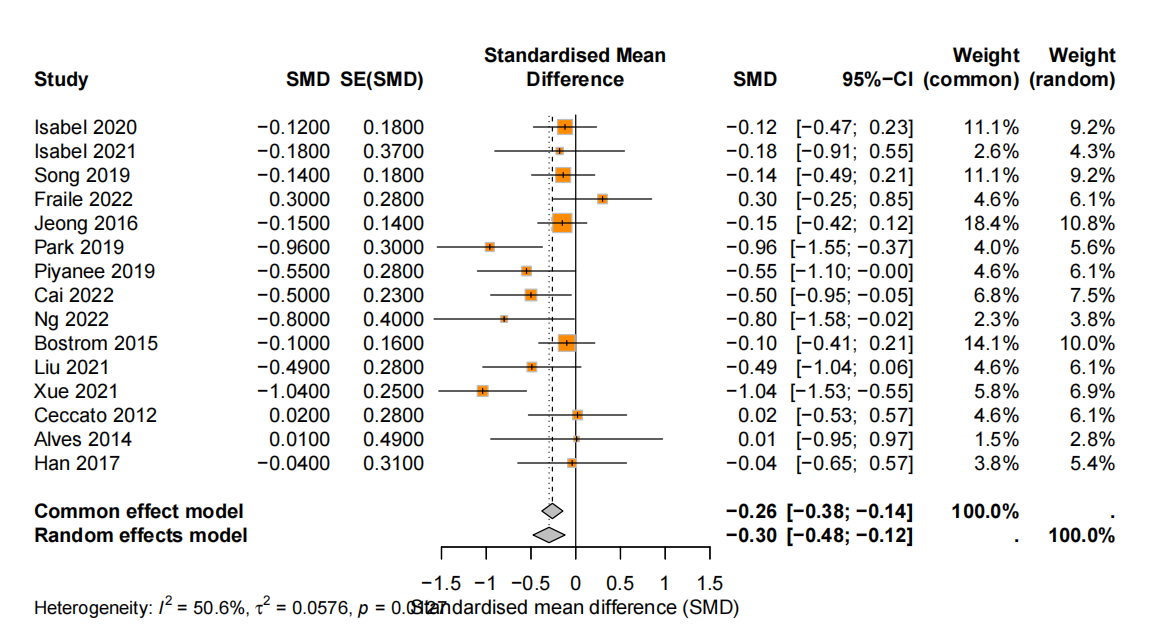  **B** |
| --- | --- |
| **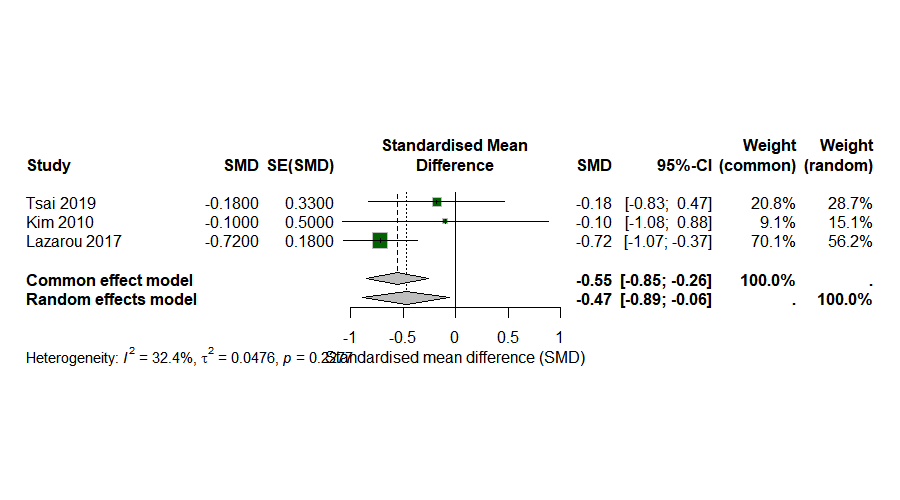**  **C** |  |

**Supplementary Figure 1** Subgroup Analyses by Depression Scale. A) Subgroup CSDD; B) Subgroup GDS; C) Subgroup BDI


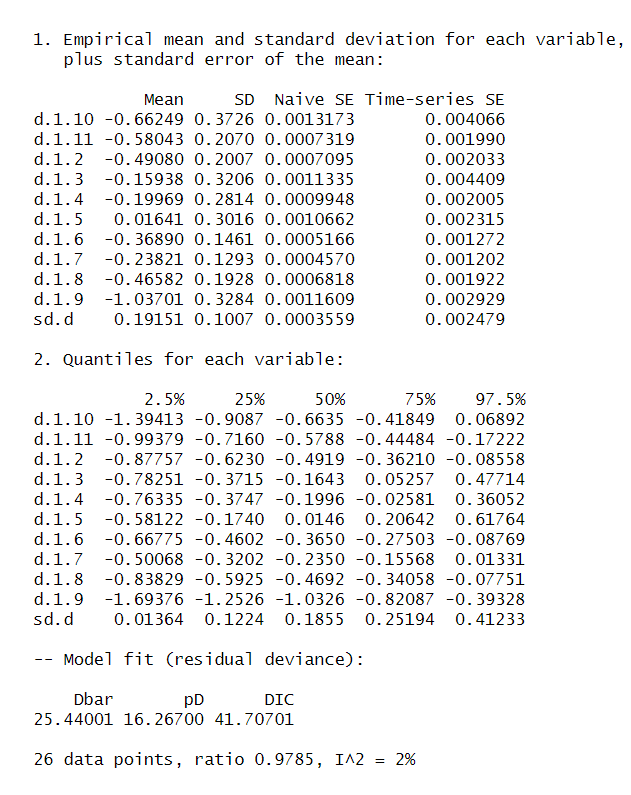


**Supplementary Figure 2** Results of network meta-analysis


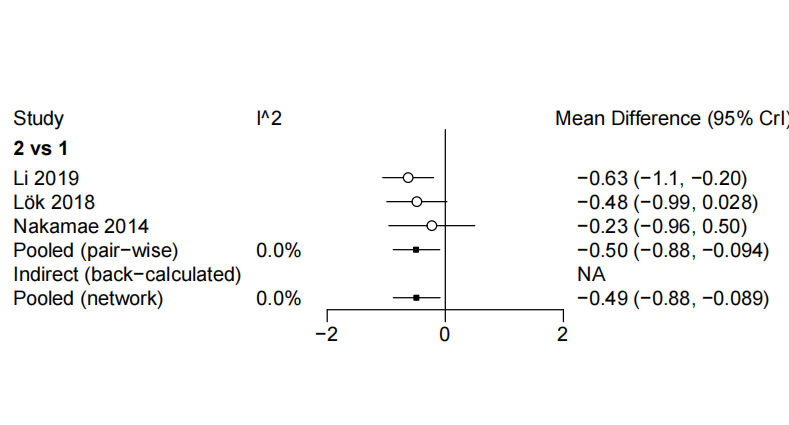


**Supplementary Figure 3** Comparative Analysis between trt 2 versus CTRL


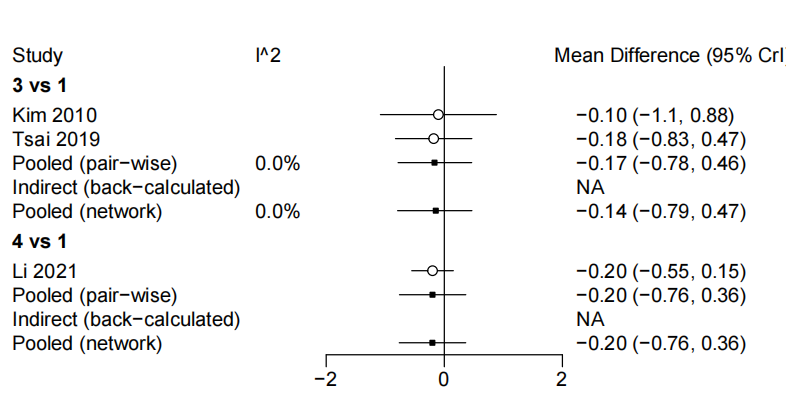
 **Supplementary Figure 4** Comparative Analysis between trt 3, 4 versus CT


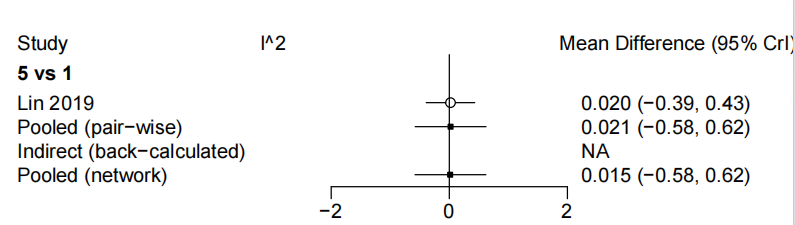


**Supplementary Figure 5** Comparative Analysis between trt 5 versus CTRL


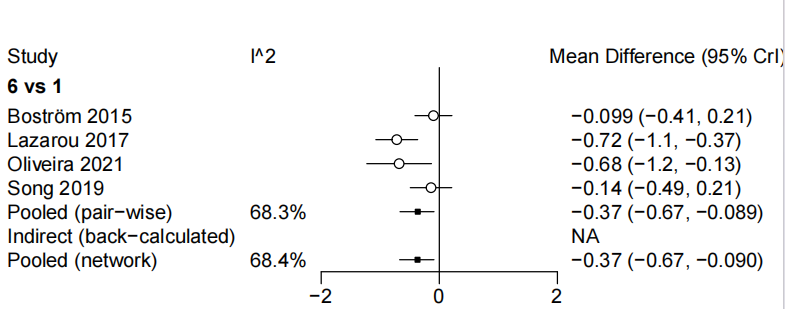


**Supplementary Figure 6** Comparative Analysis between trt 6 versus CTRL


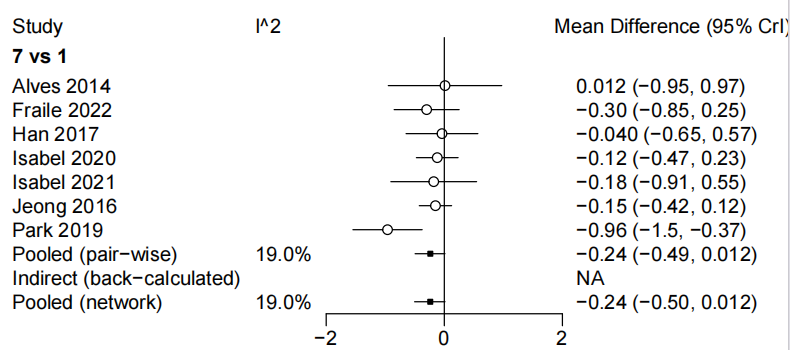


**Supplementary Figure 7** Comparative Analysis between trt 7 versus CTRL


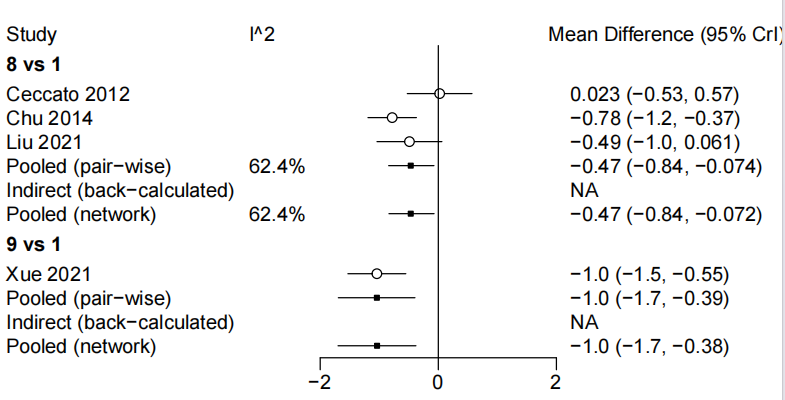


**Supplementary Figure 8** Comparative Analysis between trt 8 and 9 versus CTRL


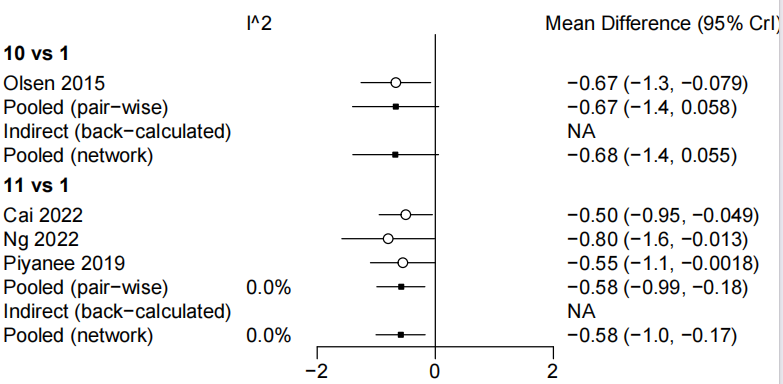


**Supplementary Figure 9** Comparative Analysis between trt 11 and 12 versus CTRL


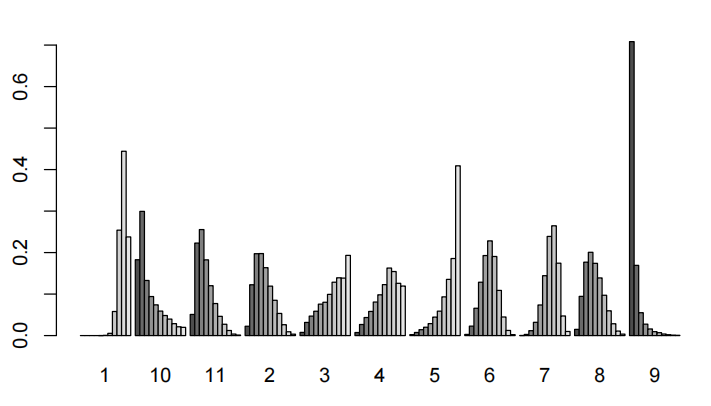


**Supplementary Figure 10** The figure of ranking probability of reduction of depression scores.


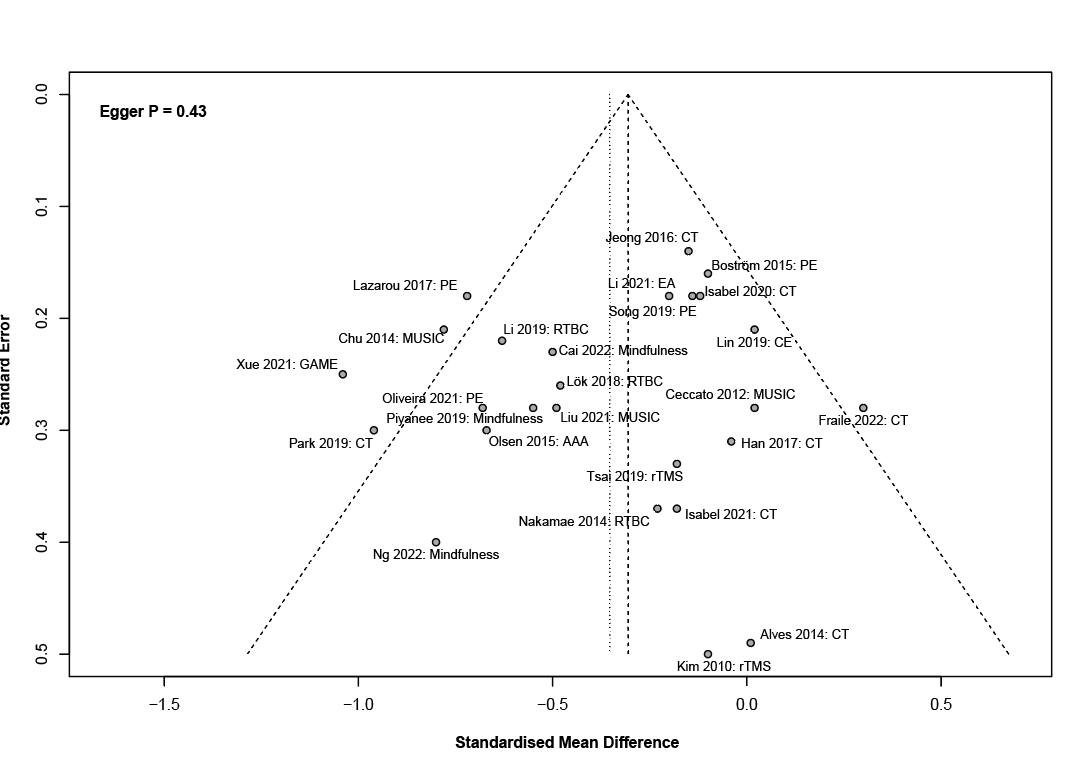


**Supplementary Figure 11** Funnel plots

| 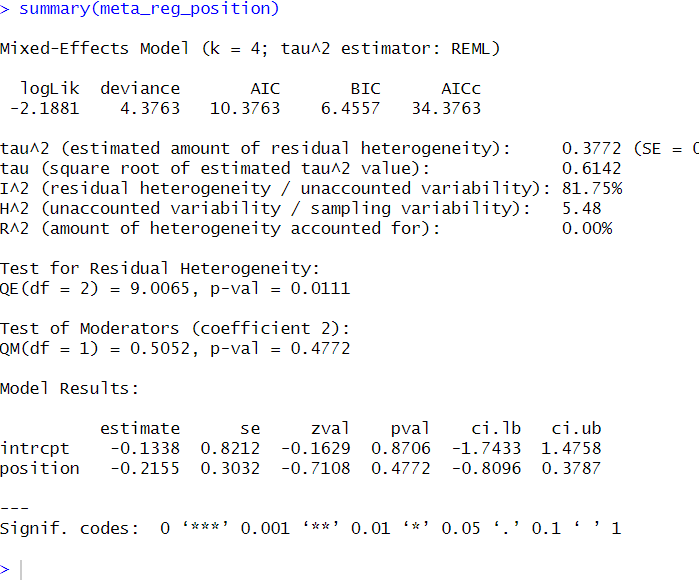  C | **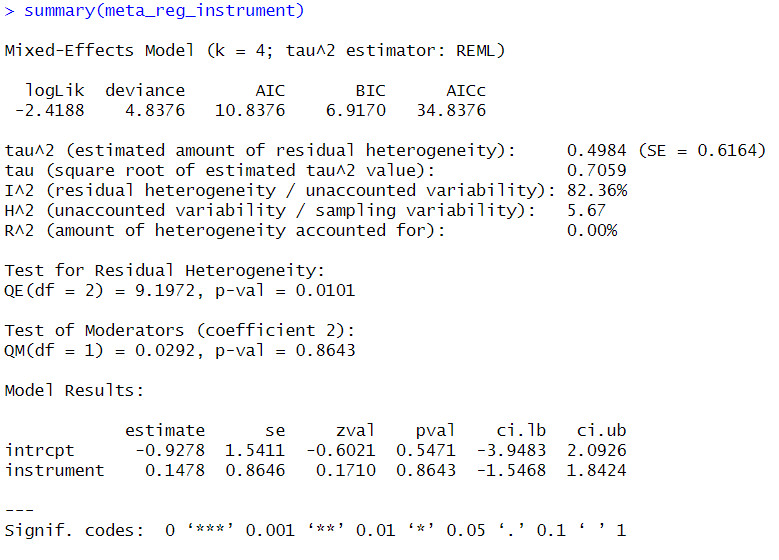**  D |
| --- | --- |
| 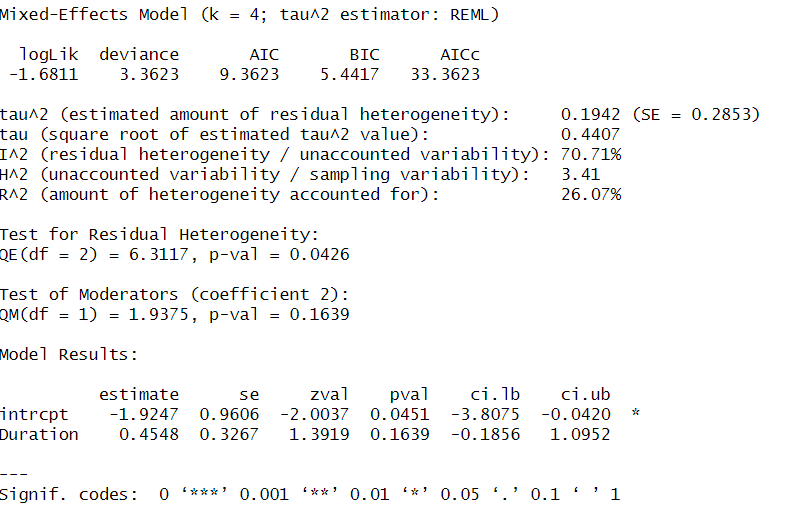 | 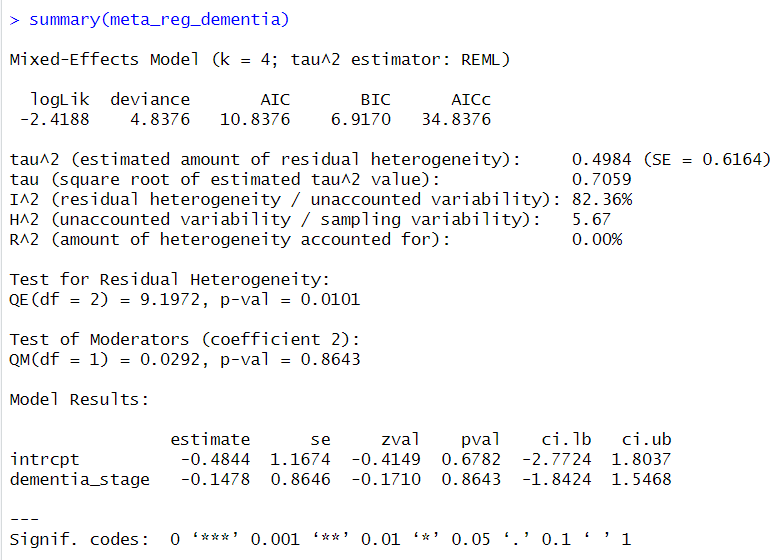 |

**Supplementary Figure 12** MUSIC Sensitivity Analyses. A) Intervention position regression; B) Instrument regression; C) Duration meta regression; D) Dementia stage regression.

B

A

| 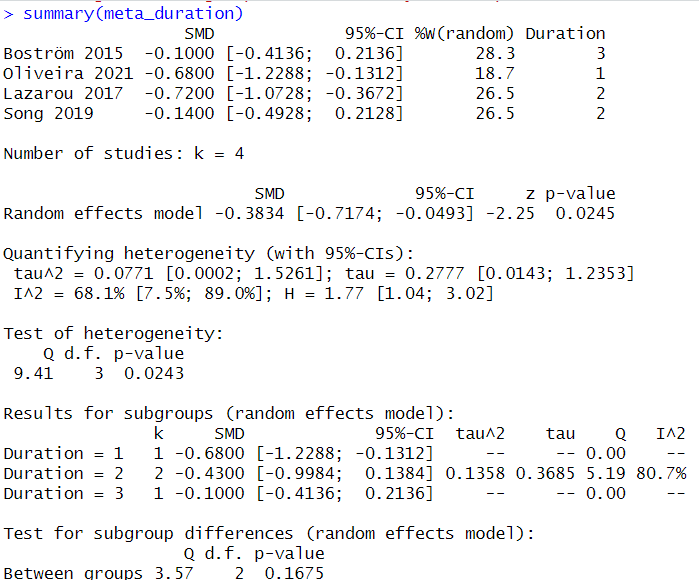  **C** | 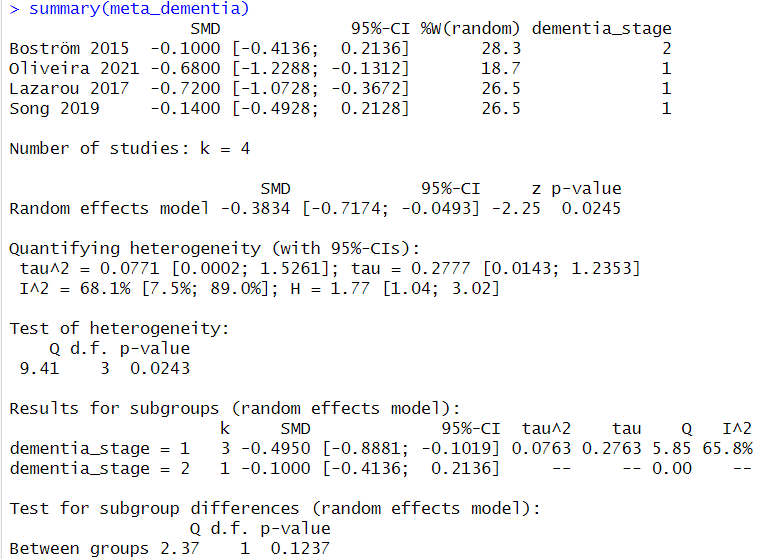 |
| --- | --- |
| 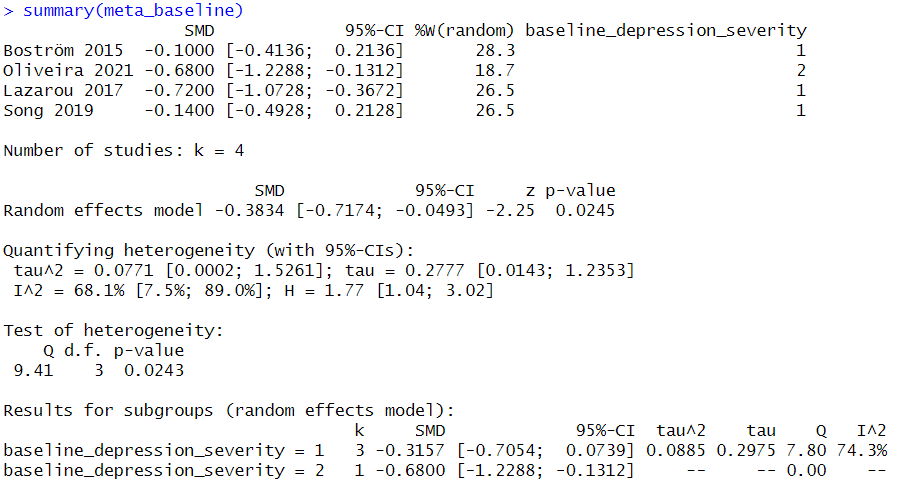 |  |

**Supplementary** **Figure 13** PE sensitivity Analyses A) Duration meta regression; B) Dementia stage regression; C) Depression severity regression

**A**

**B**

| 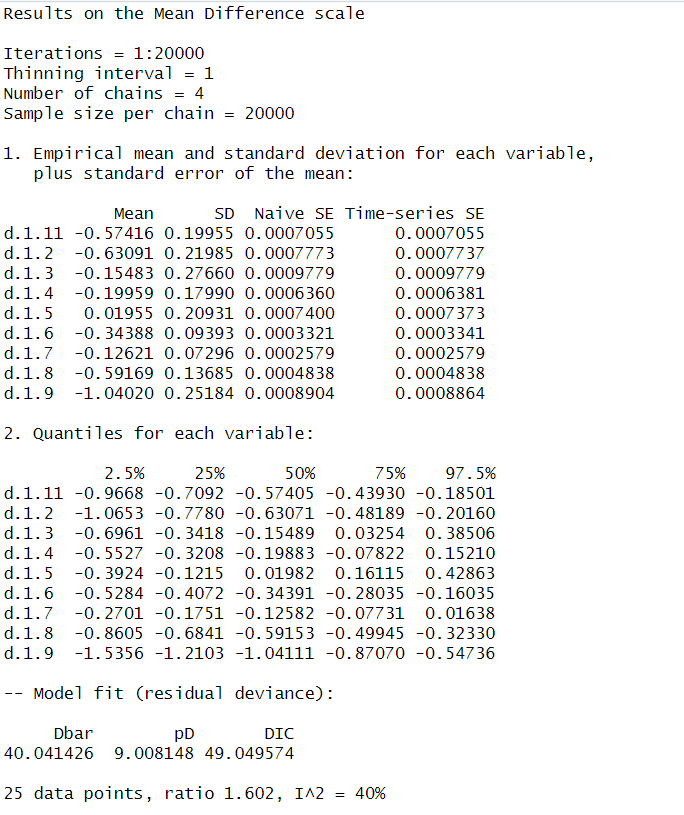  **A** | 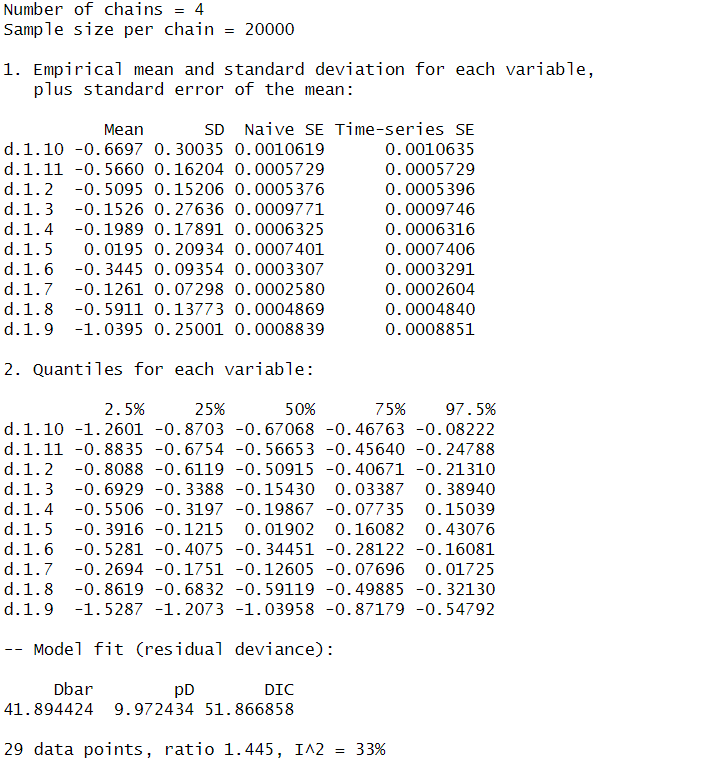  **B** |
| --- | --- |

**Supplementary Figure 14** Before and after removing high-risk articles
